# The impact of socio-economic disparities on kidney transplant outcomes: insights from a monocentric Italian study

**DOI:** 10.1007/s13304-025-02491-9

**Published:** 2025-12-19

**Authors:** Quirino Lai, Fabio Melandro, Luca Poli, Mario Piazzolla, Fatima Della Pietra, Veronica Zullino, Giulia Diamantini, Matteo Brisciani, Silvia Quaresima, Massimo Rossi, Francesco Pugliese, Manuela Garofalo

**Affiliations:** 1 General Surgery and Organ Transplantation Unit, Sapienza University of Rome, Rome, Italy; 2 Department of Anesthesiology, Critical Care Medicine and Pain Therapy, Sapienza University of Rome, Rome, Italy; 3 General Surgery and Organ Transplantation Unit, Department of General and Specialty Surgery, Sapienza University of Rome, AOU Policlinico Umberto I Rome, Viale del Policlinico 155, 00161 Rome, Italy

**Keywords:** Deprivation index, Waiting list, Living donor, Post-transplant care, Regional disparities

## Abstract

Kidney transplantation (KT) is the preferred treatment for end-stage renal disease (ESRD), but socio-economic disparities significantly influence access to transplantation, waiting list (WL) outcomes, and post-transplant results. This study evaluates the impact of the socio-economic deprivation index (SEDI) on WL and KT outcomes in an Italian center. This monocentric, retrospective cohort study analyzed 1560 adult patients enlisted for KT at Sapienza University of Rome (2000–2024). Socio-economic and clinical data were collected, and patients were stratified into low-SEDI and high-SEDI groups. The primary outcome was a composite of death and WL drop-out due to clinical worsening. Secondary outcomes included post-transplant graft loss. Kaplan–Meier survival analysis and Cox regression models were used to evaluate risk factors. Patients in high-SEDI areas had longer times from dialysis initiation to WL inscription (27 vs. 20 months, *P* < 0.001) and from WL inscription to transplantation (22 vs. 17 months, *P* < 0.001). The composite outcome of death or WL drop-out occurred in 8.7% of patients, with no significant differences between SEDI groups. However, high-SEDI patients faced significantly higher risks of post-transplant graft loss due to socio-economic factors, including vulnerable housing conditions and demographic disparities. Multivariable Cox analysis identified dialysis-to-WL duration and socio-economic factors as significant predictors of WL and post-transplant outcomes. Socio-economic factors, as measured by SEDI, significantly influence WL and KT outcomes, underscoring the need for targeted interventions to minimize delays and improve access in high-SEDI regions. Strategies such as early referral, live donor promotion, and equitable healthcare access are crucial for optimizing KT outcomes.

## Introduction

Kidney transplantation (KT) is the preferred treatment for end-stage renal disease (ESRD) due to its superior ability to enhance survival and quality of life compared to dialysis [1]. However, socio-economic factors have emerged as critical determinants of health outcomes, influencing access to transplantation, time on the waiting list (WL), and post-transplant outcomes [2–4].

The socio-economic deprivation index (SEDI), an indicator reflecting material and social deprivation, has been widely utilized to assess disparities in healthcare [5–7]. Numerous studies have highlighted significant regional disparities in socio-economic conditions across various countries, which impact multiple areas of healthcare, including KT outcomes [8, 9].

In Italy, regional discrepancies have also been observed in terms of healthcare system performance [10]. However, no studies have specifically investigated the impact of socio-economic disparities in the context of KT.

The objective of this study was to evaluate the influence of SEDI on patients enlisted for KT in an Italian center, focusing on the risk of death or drop-out due to clinical worsening during the waiting period. A secondary aim was to examine the impact of socio-economic parameters in a subgroup of transplanted patients.

## Materials and methods

This study was approved by the local ethics board of Sapienza University of Rome, Italy. Informed consent for data management was obtained from all patients at the time of WL inscription and KT. Given its retrospective nature, no additional consent was required. The Strengthening the Reporting of Observational Studies in Epidemiology (STROBE) guidelines were followed.

### Study design, setting, and population

This monocentric, retrospective cohort study analyzed data from adult patients (≥ 18 years) enlisted for KT due to chronic ESRD. The cohort included 1,560 patients enlisted between January 1, 2000, and January 31, 2024. A total of 978 (62.7%) patients were transplanted during this period, of whom 644 (41.3%) underwent transplantation at the Sapienza center. No exclusion criteria were applied.

### Outcomes, data collection, and definitions

According to the Italian Centro Nazionale Trapianti (CNT) guidelines, each KT candidate may be enlisted in two transplant centers located in different regions (https://trapianti.sanita.it/statistiche/dettaglio_organo.aspx). Consequently, some patients enlisted at Sapienza were transplanted elsewhere, and some transplanted at Sapienza were followed up only during the initial post-transplant period (3–6 months) before being transferred to centers closer to their residence.

The primary outcome was a composite event of “death” and “clinical worsening leading to WL drop-out” during the KT waiting period. Secondary outcomes included graft loss after KT, defined as return to dialysis or a serum creatinine clearance < 15 ml/min/1.73 m^2^ at the last evaluation. Patient death with a functioning graft was not censored but considered an event of interest. The last follow-up date was January 31, 2024.

The SEDI was derived from 2011 Italian census data (https://www.istat.it/en/microdata/15-italian-general-censuses-of-population-and-housing-2011-1-sample/), updated in 2020, and calculated at the census block level [11, 12]. This index assesses deprivation using five conditions: 1) low educational level; 2) unemployment; 3) living in rental property; 4) crowded housing; and 5) single-parent households with minor children. Each condition was scored binomially (0, 1), and the cumulative index ranged from 0 to 5, with higher scores indicating greater deprivation.

An arbitrary cutoff of 50% of individuals with very high SEDI risk within each province was used to stratify the population into low-SEDI (< 50%) and high-SEDI (≥ 50%) groups. The SEDI value used corresponded to the province of the patient’s dialysis center. Data were retrospectively extracted from the prospectively maintained Sapienza University database, with quality checks and single-imputation methods for handling missing data.

### Statistical analysis

Continuous variables were reported as medians and interquartile ranges (Q1–Q3), while categorical variables were expressed as counts and percentages. Comparisons were conducted using the Mann–Whitney U test for continuous variables and Chi-square or Fisher’s exact tests for categorical variables. Survival probabilities were estimated using the Kaplan–Meier method and compared with the log-rank test.

Multivariable Cox regression analyses were performed to explore the impact of clinical, transplant-related, and socio-economic variables on the risks of a) death or WL drop-out and b) graft loss post-KT. A backward Wald exclusion method was used to create parsimonious models. Hazard ratios (HRs) with 95% confidence intervals (CIs) were presented. All analyses were two-tailed, with *P*-values < 0.05 considered significant. SPSS version 27.0 (SPSS Inc., Chicago, IL, USA) was used for analyses.

## Results

Clinical- and transplant-related characteristics and socioeconomic-related characteristics of the low-SEDI (n = 745, 47.8%) and high-SEDI (n = 815, 52.2%) groups are summarized in Table 1 and Table 2, respectively.

**Table 1:** Clinical and transplant-related characteristics of the low-SEDI and high-SEDI groups

Variable	Low-SEDI (n = 745, 47.8%)	High-SEDI (n = 815, 52.2%)	*P*
	Median (Q1-Q3) or n (%)		
Male sex	484 (65.0)	509 (62.5)	0.32
*Blood group*			
0	371 (49.8)	397 (48.7)	0.24
A	232 (31.1)	280 (34.4)	
B	109 (14.6)	115 (14.1)	
AB	33 (4.4)	23 (2.8)	
No Italian citizen	86 (11.5)	28 (3.4)	< 0.001
*Age, years*			
start dialysis	48 (37–57)	46 (35–53)	< 0.001
WL inscription	51 (42–61)	50 (40–57)	0.001
at delisting	54 (44–63)	52 (43–60)	0.005
*Time, months*			
from dialysis beginning to WL inscription	20 (12–47)	27 (14–64)	< 0.001
from dialysis beginning to delisting	45 (25–89)	61 (34–104)	< 0.001
Pre-emptive at WL inscription	20 (2.7)	4 (0.5)	< 0.001
Opportunity to have a LDKT	52 (7.0)	22 (2.7)	< 0.001
WL duration, months	17 (6–37)	22 (10–42)	< 0.001
*Delisting*			
Death	55 (7.4)	58 (7.1)	0.78
Clinical worsening	10 (1.3)	13 (1.6)	
KT	476 (63.9)	502 (61.6)	
Other reasons	113 (15.2)	142 (17.4)	
Still in the list	91 (12.2)	100 (12.3)	
*PRA max value, %*			
I Class	0 (0–0)	0 (0–0)	0.65
II Class	0 (0–0)	0 (0–0)	0.35

SEDI, socio-economic deprivation index; WL, waiting list; LDKT, living-donor kidney transplantation; KT, kidney transplantation; PRA, panel reactive antibody

**Table 2:** Socio-economic characteristics of the low-SEDI and high-SEDI groups

Variable	Low-SEDI (n = 745, 47.8%)	High-SEDI (n = 815, 52.2%)	*P*
	Median (Q1-Q3) or n (%)		
*Geographical area*			
North	9 (1.2)	1 (0.1)	
Center	595 (79.9)	12 (1.5)	< 0.001
South	113 (15.2)	752 (92.3)	
Isles	28 (3.8)	50 (6.1)	
*Province population, habitants*			
0–500,000	200 (26.8)	46 (5.6)	< 0.001
500,001–999,999	79 (10.6)	153 (18.8)	
1,000,000–1,999,999	0 (-)	283 (34.7)	
2,000,000–3,999,999	6 (0.8)	333 (40.9)	
≥ 4,000,000	460 (61.7)	0 (-)	
Demographic density	745.3 (156.8–745.3)	322.9 (220.6–2591.3)	< 0.001
Rate of masculinity	90.8 (90.8–95.1)	94.8 (93.4–94.8)	< 0.001
Incidence of < 6-year-old habitants, %	5.8 (5.4–5.8)	5.9 (5.5–6.5)	< 0.001
Incidence of ≥ 75-year-old habitants, %	9.7 (9.7–10.3)	8.7 (6.7–9.3)	< 0.001
Index of dependent aged people	30.8 (30.8–30.8)	27.3 (22.6–27.4)	< 0.001
Index of dependent young people	21.3 (20.7–21.3)	23.6 (21.8–25.3)	< 0.001
Index of senescence	144.3 (144.3–144.9)	118.1 (89.1–125.4)	< 0.001
Incidence of resident strangers	85.7 (56.6–85.7)	23.1 (23.1–27.5)	< 0.001
Average size of families, n	2.3 (2.3–2.5)	2.7 (2.7–2.9)	< 0.001
Average size of families > 2.5	60 (7.4)	755 (92.6)	< 0.001
Incidence of home ownership, %	71.9 (71.9–72.7)	68.7 (56.8–71.7)	< 0.001
Mean surface of occupied houses, %	91.7 (91.7–99.1)	92.5 (89.6–98.8)	< 0.001
Incidence of buildings in a poor state of conservation, %	1.4 (1.4–1.4)	2.0 (1.5–2.0)	< 0.001
Housing crowding index	0.8 (0.5–0.8)	1.3 (0.8–2.6)	< 0.001
Incidence of illiterates, %	0.5 (0.5–1.0)	1.7 (1.7–1.9)	< 0.001
Incidence of adults with a higher education qualification, %	67.9 (55.4–67.9)	46.8 (46.8–50.2)	< 0.001
Incidence of young persons with university instruction, %	30.0 (23.4–30.0)	18.1 (17.9–22.0)	< 0.001
Unemployment rate, %	10.4 (10.4–14.2)	20.2 (18.2–26.2)	< 0.001
Employment rate, %	47.4 (42.5–47.4)	35.3 (32.7–37.6)	< 0.001
Incidence of population in very vulnerable municipalities (SEDI), %	9.3 (9.3–11.6)	90.8 (69.5–99.7)	< 0.001
Incidence of improper accommodations, %	0.3 (0.3–0.3)	0.4 (0.3–0.4)	< 0.001
Incidence of large families, %	1.1 (1.1–1.4)	2.3 (1.4–3.5)	< 0.001
Incidence of families with potential economic difficulties, %	2.4 (2.4–2.6)	5.4 (4.7–9.7)	< 0.001
Incidence of population in crowded conditions, %	1.8 (1.1–1.8)	2.7 (1.7–5.0)	< 0.001
Incidence of young people outside the job market and training, %	11.6 (11.6–12.0)	19.2 (16.9–22.4)	< 0.001
Incidence of families with potential hardship in care, %	2.5 (2.5–3.0)	2.8 (1.8–3.0)	< 0.001

SEDI, socio-economic deprivation index

During a median WL period of 19 months (Q1-Q3 = 8–40), a global number of 1,369 (87.8%) patients were delisted, while 191 (12.2%) cases were still on the list at the time of last follow-up date.

Looking in detail at the reasons for delisting, 113 (7.2%) died and 23 (1.5%) dropped-out due to clinical worsening. In the great majority of cases, the clinical worsening was caused by a multifactual deterioration of the clinical conditions (n = 17/23, 73.9%), followed by specific clinical causes involving the liver (n = 2, 8.7%), hearth, pancreas, vascular atherosclerotic disease, and high BMI (n = 1 each, 4.3%). Therefore, 136 (8.7%) patients experienced the composite event “death + clinical worsening”.

Other reasons for delisting were reported in 255 (16.3%) cases, comprehending patient lost to follow-up (n = 121, 7.8%), patient’s decision to exit from the WL (n = 64, 4.1%), patient moved to another KT center (n = 26, 1.7%), and other unspecified causes (n = 44, 2.8%).

A total number of 978 (62.7%) patients were transplanted, of whom 74/978 (7.6%) received a LDKT. A number of 644/978 (65.8%) patients were transplanted in Sapienza center, with 57/644 (8.9%) LDKT. The remaining 334/978 (34.2%) cases were transplanted in other Italian KT centers, of whom 17/334 (5.1%) received a graft from a live donor.

As for the differences between low- and high-SEDI groups, several differences were reported in terms of clinical and transplant-related characteristics (Table 1). In detail, a higher percentage of non-Italian citizens was enlisted in areas with low SEDI (11.5 vs. 3.4%, *P* < 0.001). Overall, the patients were older in the low SEDI areas at the time of dialysis beginning (*P* < 0.001), WL inscription (*P* = 0.001), and at delisting (*P* = 0.005).

The median time passed from the dialysis beginning to the WL inscription was longer in the high SEDI Group (27 vs. 20 months, *P* < 0.001). Similarly, the WL duration time (22 vs. 17 months, *P* < 0.001) and the overall time from dialysis beginning to delisting (61 vs. 45 months, P < 0.001) were longer. Inscription in the WL as a pre-emptive (2.7 vs. 0.5%, *P* < 0.001) and the opportunity to have a live donor (7.0 vs. 2.7%, *P* < 0.001) were more common conditions in the low SEDI Group.

No substantial differences were observed in the two groups comparing the overall number of cases transplanted (63.9 vs. 61.6), died (7.4 vs. 7.1%), or dropped-out for clinical worsening (1.3 vs. 1.6%) (P = 0.78).

As expected, relevant differences between the two groups were reported when the socio-economic differences were compared (Table 2). A relevant difference was reported in terms of geographical distribution, with the great majority of high-SEDI cases reported in regions located in the South Italy (92.3%, P < 0.001).

The median incidence of population living in very vulnerable municipalities according to the SEDI was lower in the low-SEDI Group (9.3 vs. 90.8%, *P* < 0.001).

As for the educational level, the median incidence of illiterates was higher in the high-SEDI Group (1.7 vs. 0.5%, P < 0.001), while the median number of persons with university grading was higher in the low-SEDI Group (30.0 vs. 18.1%, P < 0.001).

Looking at the median employment rate, it was higher in the low-SEDI Group (47.4 vs. 35.3%, *P* < 0.001), while the median unemployment rate was lower (10.4 vs. 20.2%, *P* < 0.001). the median incidence of young people outside the job market and training was higher in the high-SEDI Group (19.2 vs. 11.6%, *P* < 0.001).

As for the dimension of the families and the housing problems, the median average size of families was lower in the low-SEDI Group (2.3 vs. 2.7, *P* < 0.001). In the high-SEDI Group, more families with potential economic difficulties (median value: 5.4 vs. 2.4%, *P* < 0.001), and families with potential hardship in care (median value: 2.8 vs. 2.5%, *P* < 0.001) were observed.

The median incidence of home ownership was higher in the low-SEDI Group (71.9 vs. 68.7%, *P* < 0.001), while in the high-SEDI Group a higher median incidence of buildings in a poor state of conservation (2.0 vs. 1.4%, P < 0.001), and a higher surface of occupied houses (92.5 vs. 91.7%, *P* < 0.001).

### Risk factors for death + dropout for clinical worsening during the waiting time

Including all the clinical and socio-economic factors, a model for the risk of death-dropout during the WL period was created using a multivariable Cox regression analysis. As expected, the clinical variables played a most relevant role, with the duration of time passed from the dialysis to the listing as the most important independent risk factor (HR = 1.01, 95%CI = 1.00–1.01; *P* < 0.001). On the opposite, the longer the total dialysis duration was, the lower the risk of death-dropout (HR = 0.64, 95%CI = 0.56–0.74; *P* < 0.001), probably due to a selection-by-time process. Among the socioeconomic features, the mean surface of occupied houses (HR = 0.94, 95%CI = 0.90–0.99; *P* = 0.02) and the demographic density (HR = 0.99, 95%CI = 0.99–1.00; *P* = 0.03) were protective for the risk of death-dropout (Table 3).

**Table 3:** Risk factors for death or drop-out for clinical worsening in patients enlisted for kidney transplantation

Variable	Beta	SE	Wald	HR	Lower	Upper	*P*-value
*All the variables*							
Dialysis duration months	− 0.44	0.07	37.68	0.64	0.56	0.74	< 0.001
Duration dialysis-listing	0.007	0.00	31.95	1.01	1.00	1.01	< 0.001
PRA max value (any class)	− 0.007	0.00	7.20	0.99	0.99	0.99	0.007
Mean surface of occupied houses	− 0.06	0.03	5.39	0.94	0.90	0.99	0.02
Demographic density	− 0.001	0.00	4.90	0.99	0.99	1.00	0.03
Blood group 0	Ref	–	7.14	1.00	–	–	0.07
Blood group A	0.49	0.19	6.46	1.63	1.12	2.37	0.01
Blood group B	− 0.23	0.72	0.10	0.79	0.19	3.27	0.75
Blood group AB	0.08	0.27	0.09	1.09	0.64	1.85	0.76
Incidence of large families	0.30	0.18	2.82	1.36	0.95	1.93	0.09
Only variables connected with vulnerability*							
Demographic density	− 0.00	0.00	5.29	0.99	0.99	1.00	0.02
Mean surface of occupied houses	− 0.06	0.03	5.27	0.94	0.90	0.99	0.02
Incidence of large families	0.67	0.36	3.48	1.95	0.97	3.91	0.06
Incidence of population in very vulnerable municipalities (SEDI)	− 0.01	0.01	1.81	0.99	0.98	1.00	0.18

-2Loglikelihood = 1518.93; -2log likelihood 1581.54*

SE, standard error; HR, hazard ratio; PRA, panel reactive antibody; SEDI, socio-economic deprivation index

Only considering the socio-economic features for constructing the model, the demographic density (HR = 0.99, 95%CI = 0.99–1.00; *P* = 0.02) and the mean surface of occupied houses (HR = 0.94, 95%CI = 0.90–0.99; *P* = 0.02) confirmed their protective relevance: the higher their values, the lower the risk of death or drop-out.

The incidence of large families and the incidence of population in very vulnerable municipalities according to the SEDI only merged the statistical relevance (Table 3).

### Risk factors for graft loss after transplantation

Considering the 644 patients transplanted in the Sapienza center, two different multivariable Cox regression analyses were performed with the intent to investigate the risk factors for 90-day and overall graft loss after transplantation (Table 4).

**Table 4:** Risk factors for 90-day and overall graft loss after kidney transplantation

Variable	Beta	SE	Wald	HR	Lower	Upper	P-value
*90-day graft loss*							
Patient age at KT	0.05	0.02	9.90	1.05	1.02	1.08	0.002
Blood group 0	Ref	-	9.11	1.00	–	–	0.03
Blood group A	1.06	0.61	2.99	2.89	0.87	9.59	0.08
Blood group B	− 0.18	0.73	0.06	0.83	0.20	3.49	0.80
Blood group AB	1.30	0.82	2.53	3.66	0.74	18.16	0.11
Overall graft loss*							
Patient age at KT	0.05	0.01	37.01	1.05	1.03	1.06	< 0.001
Incidence of buildings in a poor state of conservation	1.78	0.55	10.34	5.88	2.00	16.67	0.001
Incidence of < 6-year-old habitants	3.89	1.27	9.42	49.05	4.08	589.51	0.002
Incidence of ≥ 75-year-old habitants	0.87	0.32	7.53	2.38	1.28	4.44	0.006
Incidence of resident strangers	0.04	0.02	5.80	1.04	1.01	1.08	0.02
Time between dialysis and KT > 12 months	0.97	0.42	5.30	2.63	1.15	5.97	0.02
Incidence of population in very vulnerable municipalities (SEDI)	− 0.03	0.01	4.22	1.03	1.01	1.05	0.04

-2Loglikelihood = 428.01; -2log likelihood 1624.51*

SE, standard error; HR, hazard ratio; KT, kidney transplantation; SEDI, socio-economic deprivation index

Looking at the risk factors for early (≤ 3-month) graft loss, no socio-economic aspects raised as specific risk factors for graft loss. In detail, the age of the recipient (HR = 1.05, 95%CI = 1.02–1.08; *P* = 0.002) and the different AB0 blood groups (*P* = 0.03) were relevant independent predictors for graft failure.

On the opposite, when the overall risk of graft loss was explored, several socio-economic aspects raised as relevant factors. In detail, patient age confirmed its detrimental effect (HR = 1.05, 95%CI = 1.03–1.06; *P* < 0.001). Similarly, time passed between the beginning of dialysis and KT > 12 months (HR = 2.63, 95%CI = 1.15–5.97; P = 0.02) was a risk factor for graft loss. Among the socio-economic aspects, the incidence of population in very vulnerable municipalities according to the SEDI (HR = 1.03, 95%CI = 1.01–1.05; *P* = 0.04), the incidence of buildings in a poor state of conservation (HR = 5.88, 95%CI = 2.00–16.67; *P* = 0.001), the incidence of < 6-year-old (HR = 49.05, 95%CI = 4.08–589.51; *P* = 0.002) and ≥ 75-year-old habitants (HR = 2.38, 95%CI = 1.28–4.44; *P* = 0.006), and the incidence of resident strangers (HR = 1.04, 95%CI = 1.01–1.08; *P* = 0.02) resulted as risk factors for graft failure.

### Survival curves

**Fig. 1:**
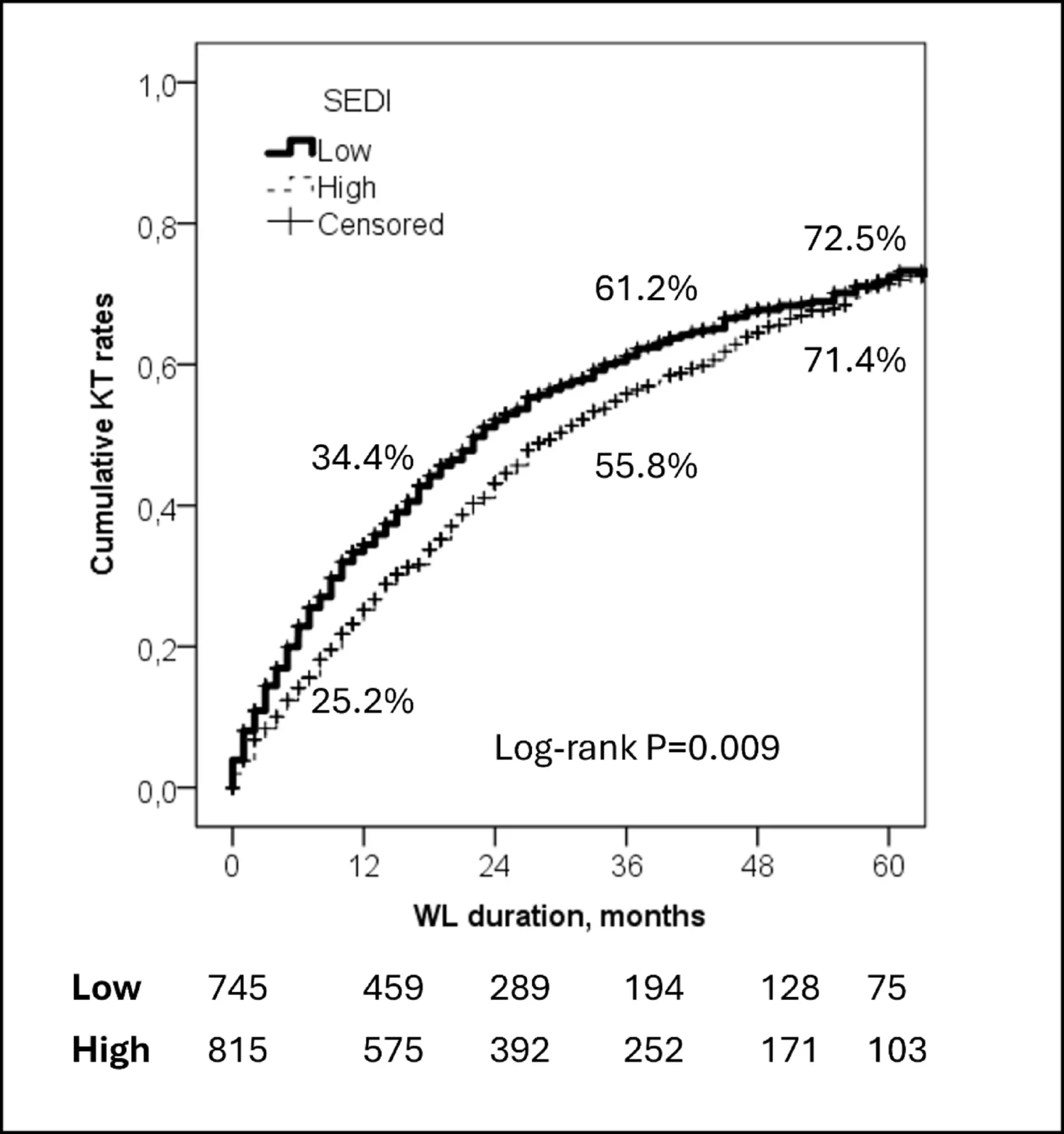
Cumulative kidney transplantation rates in the patients with low vs. high SEDI

Looking at the cumulative rates of KT in the patients enlisted for transplantation, patients in the low-SEDI Group were more rapidly transplanted, with a 1-, 3-, and 5-year rate of KT of 34.4, 61.2, and 72.5% vs. 25.2, 55.8, and 71.4% observed in the high-SEDI group, respectively (Log-rank *P* = 0.009) (Fig. 1).

**Fig. 2:**
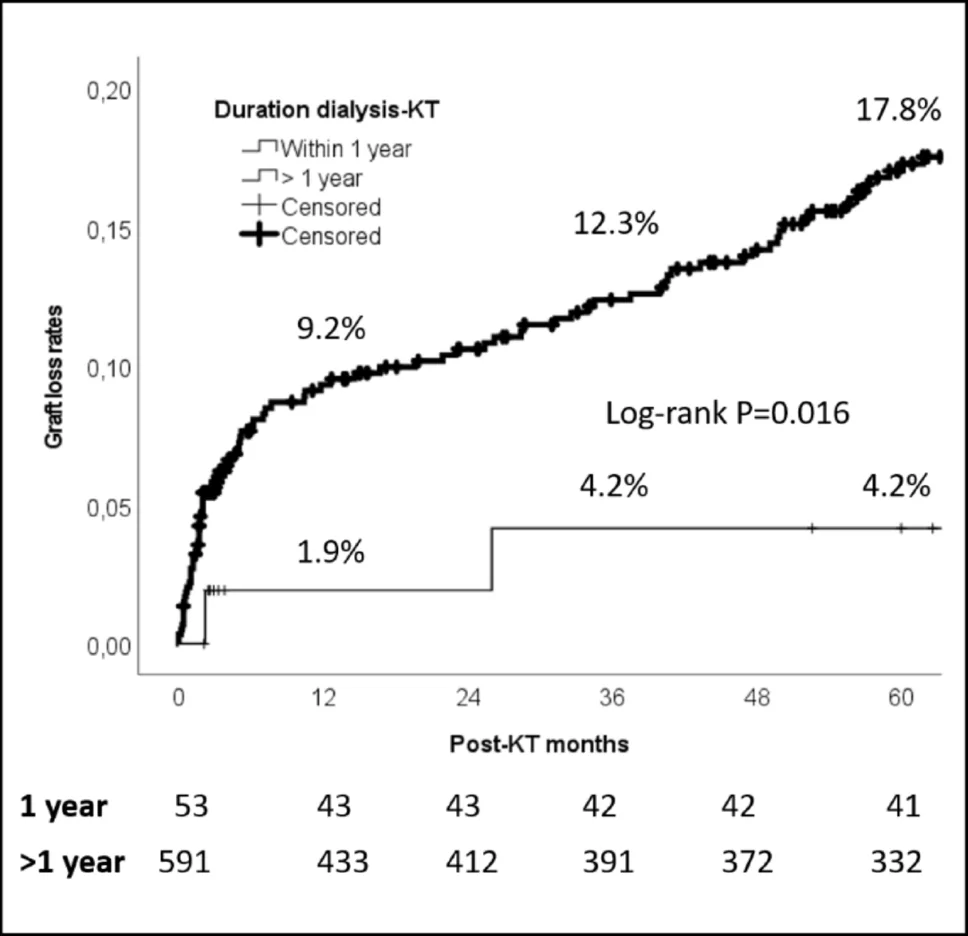
Post-KT graft survival in patients with short (≤ 1 year) vs. long (> 1 year) period between beginning of dialysis and transplantation

Performing a sub-analysis focused on the 644 patients transplanted in the Sapienza center, the possibility to be more rapidly transplanted showed a relevant impact also in terms of post-KT results. In detail, patients with a short period (< 12 months) between the beginning of dialysis and KT had excellent survivals after transplant, with 1-, 3-, and 5-year non-censored graft loss rates of 1.9, 4.2, and 4.2%. On the opposite, a longer period between dialysis starting and transplant negatively impacted (1-, 3-, and 5-year non-censored graft loss rates: 9.2, 12.3, and 17.8%; log-rank *P* = 0.016) (Fig. 2).

## Discussion

The present study highlights the significant influence of socio-economic factors, as measured by SEDI, on the outcomes of patients listed for KT. While clinical factors remain the primary determinants of WL outcomes, our findings underscore the importance of socio-economic conditions in shaping both WL and post-transplant trajectories.

Patients from high-SEDI areas experienced longer delays from the initiation of dialysis to WL inscription and longer durations on the WL. These findings align with prior studies reporting similar results. A prospective, observational cohort study from United Kingdom kidney centers explored preemptive listing and listing within two years of starting dialysis by center, identifying lower socioeconomic status as a factor associated with a lower likelihood of being listed [13].

Our findings therefore argue for structured, patient‑centred educational programs that explicitly target disadvantaged populations. Such programs should be literacy‑adapted, culturally sensitive, and geared toward empowerment in shared decision‑making along the wait‑listing pathway [14].

A French study on 11,655 incident dialysis patients eligible for KT list registration reported that high SEDI directly impacted a lower registration rate (OR = 0.82) and indirectly influenced outcomes through emergency-start dialysis (OR = 0.97), hemoglobin < 11 g/dL and/or lack of erythropoietin (OR = 0.96), and albumin < 30 g/L (OR = 0.98) [15]. These delays likely stem from systemic inequities in healthcare access and awareness, reflecting disparities in healthcare infrastructure between regions.

The duration from dialysis initiation to WL inscription emerged as a key risk factor for death or WL drop-out, with longer durations correlating with increased risk. This emphasizes the need for early referral and expedited WL inscription, particularly in socio-economically disadvantaged areas. Protective socio-economic factors, such as higher demographic density and larger housing surfaces, further highlight the complex interplay between socio-economic variables and WL outcomes.

Within this framework, education and navigation support are not ancillary but foundational: tailored counselling, simplified written/visual materials, and interpreter‑mediated sessions may reduce delays from dialysis initiation to WL inscription, particularly in high‑SEDI areas. Educational attainment may also function as a pragmatic proxy for health literacy and digital engagement, potentially shaping patients’ ability to navigate WL processes and access LDKT opportunities [16].

Minimizing the time from dialysis initiation to WL inscription and from WL inscription to KT is vital, and having a live donor could play a critical role. In our analysis, the percentage of potential live donors was notably lower among patients from high-SEDI areas, reinforcing the association between high SEDI and reduced confidence and ability to navigate healthcare systems. Several studies support this phenomenon.

Although the number of LDKT cases in our cohort was limited, the well‑established survival advantages of LDKT suggest that expanding equitable access to LDKT could partially mitigate the adverse impact of socio‑economic deprivation on graft outcomes [17].

In our center, the LDKT pathway is typically clinician‑initiated, yet motivated patients and families often act as key drivers. It is plausible that lower SEDI is associated with higher digital literacy, stronger family networks, and greater self‑advocacy, which collectively facilitate LDKT access; conversely, high‑SEDI patients may face barriers spanning awareness, logistics, and social support. Future qualitative or mixed‑method research is warranted to elucidate these mechanisms and inform targeted interventions [18].

A UK study involving 32 deceased-donor transplant recipients explored barriers to live donation, identifying six key themes distinguishing individuals from areas of high and low SEDI: passivity, disempowerment, lack of social support, short-term focus, financial concerns, and location of donors [18]. Another UK monocentric study of 2,040 consecutive kidney-alone transplant recipients showed a decline in live donations with increasing SEDI quintiles, from 50.4% (lowest SEDI quintile) to 27.6% (highest SEDI quintile) (*P* < 0.001). Socioeconomic deprivation (P < 0.001) and ethnicity (*P* = 0.005) were significant predictors of LDKT in multivariable analysis [19].

Post-transplant outcomes were also significantly associated with socio-economic factors. Patients from high-SEDI areas faced higher risks of graft loss, potentially due to poorer access to post-transplant care, limited health literacy, and barriers to medication adherence. Specific risk factors, such as the prevalence of vulnerable populations and housing conditions, underscore the multifactorial nature of graft outcomes.

Digital access and literacy likely intersect with these findings: patients from high‑SEDI areas may encounter substantial barriers to engaging with online education, remote monitoring, and medication‑adherence tools. Transplant programs should therefore ensure that platforms and materials are simplified, multilingual where appropriate, and accessible regardless of patients’ digital background [20].

This evidence aligns with international research showing that socio-economic parameters influence post-KT survival. A U.S. study of 1,975 patients found lower socioeconomic status associated with decreased likelihood of preemptive transplantation (OR = 0.74; *P* = 0.03), longer dialysis durations (> 3 years; OR = 1.43; *P* = 0.046), and higher death-censored graft loss (HR = 1.36; *P* = 0.036) [21]. A Canadian study of 4,414 KT recipients showed that each Canadian dollar 10,000 increase in neighborhood median income corresponded to an 8% decline in total graft failure (HR = 0.92) [22].

Such findings extend to pediatric populations, where recipients from high-SEDI neighborhoods had a 55% higher hazard of graft loss compared to those from low-SEDI neighborhoods after adjusting for base covariates, race, ethnicity, and insurance status [23].

In our cohort, the duration between dialysis initiation and KT significantly influenced post-transplant survival, with shorter durations linked to better graft outcomes. This reinforces the importance of minimizing pre-transplant dialysis exposure, especially in high-SEDI regions where delays are more common. A Canadian study (N = 4,461) found that preemptive transplantation reduced total graft failure (HR = 0.68), while patients waiting over 6.45 years had the highest risk (HR = 1.31) [24]. Similarly, a South Korean study (N = 4117) showed higher mortality risks for deceased-donor KT across all dialysis duration groups, with the highest risk in the third tertile (HR = 11.1; *P* < 0.001) [25].

The stark geographical disparities observed in this study, with high-SEDI cases concentrated in southern Italy, call for targeted policy interventions. A recent study on the Italian health data system attributed inefficiencies to regional autonomy, with 20 regions operating independently, creating regulatory fragmentation and disparities [10]. The healthcare gap between northern and southern Italy mirrors issues in other countries with regional disparities in healthcare systems [26, 27].

An underused lever within the Italian system is integrated home care assistance (IHCA). IHCA could support high‑risk recipients with medication management, home‑based monitoring, and facilitated links to outpatient care, thereby improving follow‑up adherence and potentially narrowing post‑KT inequities in high‑SEDI settings [28].

To operationalize these goals, we propose a pragmatic educational pathway: (i) structured, stage‑specific counselling (pre‑dialysis, WL inscription, pre‑KT, early post‑KT); (ii) literacy‑adapted written and audio‑visual materials; (iii) peer‑support networks and community outreach; and (iv) basic digital‑navigation training with provision of low‑barrier telehealth options. Such initiatives should be coordinated by transplant centers in partnership with regional health authorities and patient associations to ensure scalability and sustainability. In addition, we lacked information on the interval between chronic kidney disease (CKD) diagnosis and dialysis initiation (a key proxy for timely nephrology referral and early transplant planning) which should be evaluated in future studies.

Addressing these disparities through enhanced infrastructure, improved education and employment opportunities, and reduced regional inequalities could mitigate the impact of socio-economic deprivation on KT outcomes. Integrating socio-economic factors into patient assessments could help prioritize high-risk individuals for transplantation and tailor post-transplant care [29].

The study presents some limitations. The monocenter design limits generalizability, as regional and institutional differences may yield different results elsewhere. The retrospective nature of the study introduces potential biases, such as variable selection, missing data, and misclassification of outcomes or exposures. The arbitrary 50%-cutoff for SEDI grouping simplifies stratification, potentially masking intra-group variability, but it was chosen for interpretability.

Socio-economic data for SEDI calculations, based on 2020 census data, may not reflect current conditions. Nevertheless, SEDI remains a valid surrogate for regional socio-economic conditions during patient enrollment. The study predominantly included data from Central and Southern Italy, which may overemphasize associations between high SEDI and poorer outcomes. A multicentric national analysis could provide a more comprehensive perspective.

Lastly, factors such as income, access to healthcare resources, and cultural influences were not directly analyzed due to the retrospective design. Prospective studies incorporating these aspects would enhance understanding of non-clinical factors influencing transplant outcomes.

## Conclusion

Our findings emphasize the role of socio-economic factors in shaping WL and post-transplant outcomes for KT patients. Addressing socio-economic disparities, particularly in high-SEDI regions, is essential for equitable transplantation access and improved long-term outcomes. Tailored strategies addressing both clinical and socio-economic barriers are crucial for optimizing care for this vulnerable population.

## Abbreviations

BMI: Body mass index
CI: Confidence interval
CNT: Centro Nazionale Trapianti
ESRD: End-stage renal disease
HR: Hazard ratio
KT: Kidney transplantation
LDKT: Living donor kidney transplantation
OR: Odds ratio
Q1–Q3: Quartiles 1 and 3
SEDI: Socio-economic deprivation index
STROBE: Strengthening the reporting of observational studies in epidemiology
WL: Waiting list

## Declarations

### Data availability

The datasets generated during and/or analyzed during the current study are not publicly available but are available from the corresponding author on reasonable request.
